# Supplementary material for: Repeat Detector: versatile sizing of expanded tandem repeats and identification of interrupted alleles from targeted DNA sequencing
Source: NAR Genom Bioinform. 2022 Dec 5;4(4):lqac089. doi: 10.1093/nargab/lqac089 (PMC9719798; doi:10.1093/nargab/lqac089)
Supplement: lqac089_Supplemental_Files [file lqac089_supplemental_files.zip › supp-1.pdf]

REGISTRY 2004- 2017

Registry Steering committee: G. Bernhard Landwehrmeyer (PI), Anne-Catherine Bachoud-Lévi, Anna-Rita Bentivoglio, Ida Biunno, Raphael M. Bonelli, Juliana Bronzova, Jean-Marc Burgunder, Stephen B. Dunnett, Joaquim J. Ferreira, Jan Frich, Joe Giuliano, Olivia J. Handley, Arvid Heiberg, Sergey Illarioshkin, Torsten Illmann, Jiri Klempir, Jamie Levey, Tim McLean, Jørgen E. Nielsen, Susana Pro Koivisto, Markku Päivärinta, Sven Pålhagen, Oliver Quarrell, Maria Ramos-Arroyo, Raymund A.C. Roos, Carsten Saft, Ana Rojo Sebastián, Sarah J. Tabrizi, Wim Vandenberghe, Christine Verellen-Dumoulin, Tereza Uhrova, Jan Wahlström+, Jacek Zaremba

Language coordinators: Verena Baake (formerly Rödig), Katrin Barth, Monica Bascuñana Garde, Kristina Becanovic, Tomáš Bernard, Sabrina Betz, Reineke Bos, Adrien Come, Leonor Correia Guedes, Jenny Callaghan, Selene Capodarca, Sébastien Charpentier, Wildson Vieira da Silva, Martina Di Renzo, Daniel Ecker, Ana Maria Finisterra,, Ruth Fullam, Camille Genoves, Mette Gilling, Olivia J Handley, Andrea Horta, Carina Hvalstedt, Christine Held, Hasina Hussain, Kerstin Koppers, Claudia Lamanna, Matilde Laurà, Asunción Martínez Descals, Saul Martinez-Horta, Tiago Mestre, Sara Minster, Daniela Monza, Kristina Münkel, Lisanne Mütze, Martin Oehmen, Helene Padieu, Laurent Paterski, Nadia Peppa, Susana Pro Koivisto, Beate Rindal, Dawn Rogers, Niini Røren (formerly Heinonen), Ana Salgueiro, Pavla Šašinková, Yury Seliverstov, Catherine Taylor, Erika Timewell, Jenny Townhill, Patricia Trigo Cubillo, Marleen R van Walsem, Marie-Noelle Witjes-Ané, Grzegorz Witkowski, Abigail Wright, Elizaveta Yudina, Daniel Zielonka, Eugeniusz Zielonka, Paola Zinzi

Legacy database team: Daisy De Abreu, Gunter Antoneag, Katrin Barth, Sabrina Betz, Jean-Marc Burgunder, Christine Capper-Loup, Luís Garcez Ferreira, Nilza Gonçalves, Torsten Illmann, Raquel Lobo, Jürgen Nagler-Ihlein, Eileen Neacy, Ana Teresa Santos, Jan Tuschhoff, Jennifer Ware

AUSTRIA

Graz (Medizinische Universitäts Graz, Psychiatrie): Raphael M. Bonelli, Karen Hecht, Brigitte Herranhof, Anna Holl (formerly Hödl), Hans-Peter Kapfhammer, Michael Koppitz, Sabine Lilek, Markus Magnet, Nicole Müller, Daniela Otti, Annamaria Painold, Karin Reisinger, Monika Scheibl, Helmut Schöggl, Jasmin Ullah

Innsbruck (Universitätsklinik Innsbruck, Neurologie): Eva-Maria Braunwarth, Florian Brugger, Lisa Buratti, Eva-Maria Hametner, Caroline Hepperger, Christiane Holas, Anna Hotter, Anna Hussl, Barbara Larcher, Philipp Mahlknecht, Christoph Müller, Bernadette Pinter, Werner Poewe, Eva-Magdalena Reiter, Klaus Seppi, Fabienne Sprenger, Gregor Wenning

Salzburg (Christian-Doppler-Klinik Salzburg, Universitätsklinikum der PMU, Universitätsklinik für Neurologie): Raphael M. Bonelli, Gunther Ladurner, Stefan Lilek, Daniela Sinadinosa, Wolfgang Staffen, Anna Maria Walleczek

Vienna-UNI: Christoph Linder, Walter Pirker

BELGIUM

Bierbeek: Dirk Liessens, Godelinde Calmeyn, Nele Somers, Isabelle Delvaux, Andrea Boogaerts

Bruxelles (Vrije Universiteit Brussel): Anja Flamez, Sylvie de Raedt

Bruxelles (Erasmes) : Nick Alaerts, Hichem Slama, Frédéric Supiot

Bruxelles (St-Luc) : Eric Constant, Anne-Françoise Gillardin, Marie-Claude Léonard, Christine Verellen-Dumoulin, Françoise van de Wyngaerde

Charleroi (Institut de Pathologie et de Génétique (IPG)): Michel Dupuis, Cécile Minet, Pascale Ribaï, Dominique Van Paemel, Christine Verellen-Dumoulin

Leuven (Universitair Ziekenhuis Gasthuisberg): Andrea Boogaerts, Wim Vandenberghe, Dimphna van Reijen, Petra Weckx

CZECH REPUBLIC

Olomouc (Neurologická klinika, Fakultní nemocnice Olomouc): Michaela Kaiserova, Zuzana Šenkárová

Prague (Extrapyramidové centrum, Neurologická klinika, 1. LF UK a VFN): Ondřej Bezdíček, Jiří Klempíř, Olga Klempířová, Veronika Majerová-Ibarburu, Tomáš Nikolai, Jan Roth, Irena Stárková

DENMARK

Aarhus (Aarhus University Hospital): Louise Hasselstrøm Madsen, Anette Torvin Møller

Copenhagen University Hospital (Rigshospitalet, Memory clinic): Lena Hjermind, Oda Jacobsen, Ida Unmack Larsen, Suzanne Lindquist, Jørgen Nielsen, Lisbeth Regeur, Peter Roos, Jette Stockholm, Christina Vangsted-Hansen, Tua Vinther-Jensen

Odense (Odense University Hospital): Annette Lolk, Marianne Lundsgaard, Lene Wermuth

FINLAND

Aland: Christian Andersson, Clara Nyberg, Jimmy Sundblom

Helsinki - Vaestoliitto (Department of Medical Genetics): Maarit Peippo, Marjatta Sipponen

Kuopio: Anu Bruun, Paivi Hartikainen, Seija Mäkipää, Mari Ollokainen

Oulu (Dep. of Neurology): Jaana Åman, Mikko Kärppä

Oulu (Dep. of Medical Genetics): Jaakko Ignatius, Outi Jääskeläinen, Outi Kajula, Jukka Moilanen, Aki Mustonen

Tampere (Terveystalo Healthcare Service Centre): Maire Santala

Turku-Suvituuli (Rehabilitation Centre Suvituuli): Pia Eklund, Heli Hiivola, Hannele Hyppönen, Kirsti Martikainen, Marjut Ojala, Sirkku Tähkäpää,Katri Tuuha

FRANCE

Angers (Centre de référence des maladies neurogénétique- CHU d'Angers): Philippe Allain, Dominique Bonneau, Marie Bost, Bénédicte Gohier, Marie-Anne Guérid, Audrey Olivier, Julie Prouzet, Adriana Prundean, Clarisse Scherer-Gagou, Christophe Verny

Bordeaux (Hôpital Pellegrin): Blandine Babiloni, Déborah Bled, Sabrina Debruxelles, Charlotte Duché, Sonia Fraisse, Cyril Goizet, Laetitia Jameau, Danielle Lafoucrière, Umberto Spampinato

Oberer Eselsberg 45/1 · 89081 Ulm · Germany
Phone +49 (0)731 500-63101 · Fax +49 (0)731 500-63082 · www.euro-hd.net

Clermont-Ferrand (Hôpital Gabriel Montpied): Julien Couttier, Bérengère Debilly, Christine Delaigue, Philippe Derost, Franck Durif, Véronique Germain, Perrine Legendre, Sylvie Loiseau, Ana Marques, Miguel Ulla, Tiphaine Vidal

Creteil (Hôpital Henri Mondor): Anne-Catherine Bachoud-Lévi, Farideh Badei, Marie-Françoise Boissé, Lotfi Boudali, Laurent Cleret de Langavant, Laurie Lemoine, Graca Morgado, Katia Youssov

Lille-Amiens:

Lille (CHRU Roger Salengro): Agnès Annic, Recka Barthélémy, Christelle De Bruycker, Maryline Cabaret, Anne-Sophie Carette, Nicolas Carrière, Eric Decorte, Luc Defebvre, Marie Delliaux, Arnaud Delval, Alizé Depelchin, Alain Destee, Nelly Dewulf-Pasz , Thibaut Dondaine, Florence Dugauquier, Kathy Dujardin, Lucie Hopes, Pierre Krystkowiak, Marie-Hélène Lemaire, Sylvie Manouvrier, Eugénie Mutez, Mireille Peter, Lucie Plomhause, Bernard Sablonnière, Clémence Simonin, Céline Tard, Stéphanie Thibault-Tanchou, Isabelle Vuillaume

Amiens (CHU Sud): Marcellin Bellonet, Stéphanie Blin, Simone Chen, Pierre Krystkowiak, Kamel Masmoudi, Gilles Morin, Martine Roussel, Mélissa Tir, Béatrice Schüler, Sandrine Wannepain, Yassine Zouitina

Marseille (Hôpital La Timone): Jean-Philippe Azulay, Marie Delfini, Alexandre Eusebio, Frédérique Fluchere, Aicha Guenam, Laura Mundler, Karine Nguyen

Paris (Hôpital de la Pitié Salpêtrière): Sandra Benaich, Alexis Brice, Sarah Boster, Perrine Charles, Alexandra Durr, Claire Ewenczyk, Hélène Francisque, Céline Jauffret, Damian Justo, Abdulrahman Kassar, Stephan Klebe, Fabien Lesne, Paolo Milani, Marie-Lorraine Monin, Tiffany Monnier, Emmanuel Roze, Alina Tataru, Maya Tchikviladzé

Rouen (Hôpital Charles Nicolle): Sandrine Bioux, Evangeline Bliaux, Carole Girard, Lucie Guyant-Maréchal, Didier Hannequin, Véronique Hannier, Séverine Jourdain, David Maltête, Dorothée Pouliquen

Strasbourg (Hôpital Civil): Mathieu Anheim, Nadia Barun, Ouhaid Lagha-Boukbiza, Nadine Longato, Christophe Marcel, Clélie Phillipps, Gabrielle Rudolf, Gisèle Steinmetz, Christine Tranchant, Caroline Wagner, Marie-Agathe Zimmermann

Toulouse (Hôpital Purpan): Leily Blondeau, Fabienne Calvas, Samia Cheriet, Helène Delabaere, Jean-François Demonet, Laurent Marquine, Jérémie Pariente, Michèle Pierre, Elsa Pomies, Sandrine Rolland, Corinne Souyris

GERMANY

Aachen (Universitätsklinikum Aachen, Neurologische Klinik): Christoph Michael Kosinski, Eva Milkereit, Daniela Probst, Kathrin Reetz, Christian Sass, Johannes Schiefer, Christiane Schlangen, Cornelius J. Werner

Berlin (Universitätsmedizin Berlin, Klinik und Poliklinik für Neurologie): Markus Beuth, Harald Gelderblom, Josef Priller, Harald Prüß, Eike Spruth, Silvia Thiel

Bochum (Huntington-Zentrum (NRW) Bochum im St. Josef-Hospital): Jürgen Andrich, Gisa Ellrichmann, Lennard Herrmann, Rainer Hoffmann, Barbara Kaminski, Peter Kraus, Carsten Saft, Christiane Stamm

Bremen: Christos Ganos, Lars Stubbe, Vera Tadic, Jennifer Tübing

Dinslaken (Reha Zentrum in Dinslaken im Gesundheitszentrums Lang): Herwig Lange

Dresden (Universitätsklinikum Carl Gustav Carus an der Technischen Universität Dresden, Klinik und Poliklinik für Neurologie): Cecile Bosredon, Ulrike Hunger, Matthias

Oberer Eselsberg 45/1 · 89081 Ulm · Germany
Phone +49 (0)731 500-63101 · Fax +49 (0)731 500-63082 · www.euro-hd.net

Löhle, Antonia Maass, Christiana Ossig, Simone Schmidt, Alexander Storch, Annett Wolz, Martin Wolz

Erlangen (Universitätsklinikum Erlangen, Molekulare Neurologie und Klinik für Neurologie): Zacharias Kohl, Christina Kozay, Jasmin Ullah, Jürgen Winkler

Freiburg (Universitätsklinik Freiburg, Neurologie): Ulrike Bergmann, Regina Böringer, Philipp Capetian, Gerit Kammel, Johann Lambeck, Miriam Mächtel, Simone Meier, Michel Rijntjes, Birgit Zucker

Hamburg (Universitätsklinikum Hamburg-Eppendorf, Klinik und Poliklinik für Neurologie): Kai Boelmans, Christos Ganos, Ines Goerendt, Walburgis Heinicke, Ute Hidding, Jan Lewerenz, Alexander Münchau, Michael Orth, Jenny Schmalfeld, Lars Stubbe, Simone Zittel

Hannover (Neurologische Klinik mit Klinischer Neurophysiologie, Medizinische Hochschule Hannover): Gabriele Diercks, Dirk Dressler, Flverly Francis, Sabine Gayde-Stephan, Heike Gorzolla, Bianca Kramer, Rebecca Minschke, Christoph Schrader, Pawel Tacik

Itzehoe (Schwerpunktpraxis Huntington, Neurologie und Psychiatrie): Michael Ribbat+

Marburg KPP (Klinik für Psychiatrie und Psychotherapie Marburg-Süd): Bernhard Longinus

Marburg UNI (Universitätsklinik Marburg, Sprechstunde für choreatiforme Bewegungsstörungen): Carsten Möller, Katrin Bürk

München (Huntington-Ambulanz im Neuro-Kopfzentrum - Klinikum rechts der Isar der Neurologischen Klinik und Poliklinik der Technischen Universität München): Antje Lüsebrink, Mark Mühlau, Alexander Peinemann, Michael Städtler, Adolf Weindl, Juliane Winkelmann, Cornelia Ziegler

Münster (Universitätsklinikum Münster, Klinik und Poliklinik für Neurologie): Natalie Bechtel, Heike Beckmann, Stefan Bohlen, Nicole Göpfert, Eva Hölzner, Herwig Lange, Ralf Reilmann, Stefanie Rohm, Silke Rumpf, Christian Sass, Sigrun Schepers, Nathalia Weber

Taufkirchen (Isar-Amper-Klinikum - Klinik Taufkirchen (Vils)): Michael Bachmeier, Matthias Dose, Nina Hofstetter, Ralf Marquard, Alzbeta Mühlbäck

Ulm (Universitätsklinikum Ulm, Neurologie): Katrin Barth, Andrea Buck, Julia Connemann, Daniel Ecker, Carolin Geitner, Christine Held, Andrea Kesse, Bernhard Landwehrmeyer, Franziska Lezius, Jan Lewerenz, Solveig Nepper, Anke Niess, Michael Orth, Ariane Schneider, Daniela Schwenk, Sigurd Süssmuth, Sonja Trautmann, Melanie Vogel, Patrick Weydt

Würzburg (Universitätsklinikum Würzburg, Neurologie): Stephan Klebe, Thomas Musacchio, Christine Leypold, Kerstin Nöth

ITALY

Bari (Neurophysiopathology of Pain Unit, Basic Medical, Neuroscience and Sensory System Department, University of Bari): Claudia Cormio, Olimpia Difruscolo, Giovanni Franco, Angela Nuzzi, Vittorio Sciruicchio, Claudia Serpino, Marina de Tommaso

Bologna (DIBINEM - Alma Mater Studiorum - Università di Bologna, IRCCS Istituto delle Scienze Neurologiche di Bologna): Giovanna Calandra-Buonaura, Sabina Capellari, Pietro Cortelli, Roberto Gallassi, Roberto Poda, Cesa Scaglione

Brescia (Division of Biology and Genetics, Department of Molecular and Translational Medicine & Division of Neurology, Department of Clinical and Experimental Sciences,

Oberer Eselsberg 45/1 · 89081 Ulm · Germany
Phone +49 (0)731 500-63101 · Fax +49 (0)731 500-63082 · www.euro-hd.net

University of Brescia): Chiara Agosti, Sergio Barlati, Silvia Compostella, Eleonora Marchina, Alessando Padovani

Cagliari (Movement Disorders Center, Department of Neurology, Institute of Neurology, University of Cagliari): Michela Figorilli, Francesco Marrosu, Antonella Muroni, Valeria Piras, Melisa Vacca

Florence (Department of NEUROFARBA, University of Florence & Careggi University Hospital, IRCSS " Don Gnocchi "): Elisabetta Bertini, Caterina Bartoli, Fernanda Fortunato, Elena Ghelli, Andrea Ginestroni, Claudia Mechi, Marco Paganini, Silvia Piacentini, Silvia Pradella, Anna Maria Romoli, Sandro Sorbi

Genoa (Department of Neuroscience, Rehabilitation, Ophthalmology, Genetics, Maternal and Child Health, University of Genova): Giovanni Abbruzzese, Monica Bandettini di Poggio, Giovanna Ferrandes, Paola Mandich, Roberta Marchese, Emilio Di Maria, Tiziano Tamburini

Milan (SODS Genetica delle Malattie Neurodegenerative e Metaboliche & U.O. Neurologia, Fondazione IRCCS Istituto Neurologico Carlo Besta): Alberto Albanese, Simona Castagliuolo, Anna Castaldo, Stefano Di Donato, Daniela Di Bella, Cinzia Gellera, Silvia Genitrini, Caterina Mariotti, Daniela Monza, Lorenzo Nanetti, Marta Panzeri, Dominga Paridi, Paola Soliveri, Francesca Spagnolo, Franco Taroni, Chiara Tomasello

Naples (Department of Neurosciences and Reproductive and Odontostomatological Sciences, Federico II University of Naples): Giuseppe De Michele, Luigi Di Maio, Carlo Rinaldi, Marco Massarelli, Silvio Peluso, Alessandro Roca, Cinzia Valeria Russo, Elena Salvatore, Pierpaolo Sorrentino, Tecla Tucci

Pozzilli (IS) (IRCCS Neuromed): Milena Cannella, Valentina Codella, Francesca De Gregorio, Annunziata De Nicola, Francesca Elifani, Chiara Esposito, Tiziana Martino, Irene Mazzante, Martina Petrollini, Maria Simonelli, Maurizio Vezza

IRCCS Casa Sollievo della Sofferenza, San Giovanni Rotondo: Ferdinando Squitieri

Rome (LIRH Foundation): Barbara D'Alessio, Francesca Lovo

Rome (Department of Neurology, Università Cattolica del Sacro Cuore; Institute of Translational Pharmacology & Institute of Cognitive Sciences and Technologies, National Research Council of Italy): Anna Rita Bentivoglio, Francesco Bove, Claudio Catalli, Raffaella Di Giacopo, Alfonso Fasano, Marina Frontali, Arianna Guidubaldi, Tamara Ialongo, Gioia Jacopini, Giovanna Loria, Anna Modoni, Martina Petracca, Carla Piano, Piccininni Chiara, Davide Quaranta, Silvia Romano, Francesco Soleti, Marcella Solito, Maria Spadaro, Flavia Torlizzi, Paola Zinzi

Rome (Azienda Ospedaliera Sant'Andrea; Department of Neuroscience, Mental Health and Sensory Organs (NESMOS), Faculty of Medicine and Psychology, Sapienza University of Rome; Institute of Translational Pharmacology & Institute of Cognitive Sciences and Technologies, National Research Council of Italy): Giulia Coarelli, Michela Ferraldeschi, Marina Frontali, Gioia Jacopini, Giovanni Ristori, Silvia Romano, Paola Zinzi

NETHERLANDS

Enschede (Medisch Spectrum Twente): Monique S.E. van Hout, Jeroen P.P. van Vugt, A. Marit de Weert, Marloes Verhoeven

Groningen (Polikliniek Neurologie): Meike Dekker, Jesper Klooster, Nico Leenders, Joost van Oostrom, Berry Kremer

Oberer Eselsberg 45/1 · 89081 Ulm · Germany
Phone +49 (0)731 500-63101 · Fax +49 (0)731 500-63082 · www.euro-hd.net

Leiden (Leiden University Medical Centre (LUMC)): Verena Baake, Simon J. A. van den Bogaard, Reineke Bos, Eve M. Dumas, Ellen P. ‘t Hart, Marye Hogenboom, Milou Jacobs, Caroline Jurgens, Anne Kampstra, Raymund A.C. Roos, Anne Schoonderbeek, Marie-Noëlle Witjes-Ané

Maastricht: Annelien Duits, Mayke Oosterloo, Mirella Waber

Nijmegen (Universitair Medisch Centrum St. Radboud, Neurology): Berry Kremer, Carla Verstappen

NORWAY

Bergen (Haukeland University Hospital, Dept of Medical Genetics and Olaviken Psychiatric Hospital): Ellen Økland Blinkenberg. (NKS Olaviken`s HD clinic): Erik Hauge, Hilde Tyvoll

Oslo University Hospital (Dept. of Medical Genetics, Dept. of Neurology, Dept.of Neurorehabilitation): Olaf Aaserud, Nils Olaf Aanonsen, Kathrine Bjørgo, Nancy Borgerød, Elisabeth Dramstad, Madeleine Fannemel, Jan C. Frich, Per F. Gørvell, Kathrine Haggag, Cecilie Haggag Johannessen, Arvid Heiberg, Lars Retterstøl, Oddveig Røsby, Jutta Rummel, Alma Sikiric, Bodil Stokke, Marleen van Walsem, Ragnhild Wehus

Trondheim (St. Olavs Hospital): Vibeke Arntsen, Inga Bjørnevoll, Sigrid Botne Sando, Marte Gjøl Haug, Hanna Haugan Størseth, Rune Østern, Julie Paulsen

POLAND

Gdansk (St. Adalbert Hospital, Gdansk, Medical University of Gdansk, Neurological and Psychiatric Nursing Dpt.): Artur Dziadkiewicz, Agnieszka Konkel, Ewa Narożańska, Malgorzata Nowak, Piotr Robowski, Emilia Sitek, Jaroslaw Slawek, Witold Soltan, Michal Szinwelski

Katowice (Medical University of Silesia, Katowice): Michał Arkuszewski, Magdalena Błaszczyk, Magdalena Boczarska-Jedynak, Ewelina Ciach-Wysocka, Agnieszka Gorzkowska, Barbara Jasińska-Myga, Aleksandra Kaczmarczyk, Gabriela Kłodowska – Duda, Grzegorz Opala, Monika Rudzińska, Daniel Stompel

Krakow (Krakowska Akademia Neurologii): Krzysztof Banaszkiewicz, Dorota Boćwińska, Kamila Bojakowska-Jaremek, Małgorzata Dec, Natalia Grabska, Malgorzata Krawczyk, Ewelina Kubowicz, Michalina Malec-Litwinowicz, Monika Rudzińska, Agata Stenwak, Andrzej Szczudlik, Elżbieta Szczygieł, Magdalena Wójcik, Anna Wasielewska

Poznan (Poznan University of Medical Sciences, Poland): Jacek Anioła Anna Bryl, Anna Ciesielska, Aneta Klimberg, Jerzy Marcinkowski, Husam Samara, Justyna Sempołowicz, Bartłomiej Wiśniewski, Daniel Zielonka

Warsaw-MU (Medical University of Warsaw, Neurology): Anna Gogol (formerly Kalbarczyk), Piotr Janik, Zygmunt Jamrozik, Anna Kaminska, Hubert Kwiecinski+

Warsaw-IPiN (Institute of Psychiatry and Neurology Dep. of Genetics, First Dep. of Neurology): Jakub Antczak, Katarzyna Jachinska, Wioletta Krysa, Maryla Rakowicz, Rafal Rola, Danuta Ryglewicz, Halina Sienkiewicz-Jarosz, Iwona Stępniak, Anna Sułek, Grzegorz Witkowski, Jacek Zaremba, Elzbieta Zdzienicka, Karolina Ziora-Jakutowicz

PORTUGAL

Coimbra – (Hospital Universitário de Coimbra): Cristina Januário, Filipa Júlio

Oberer Eselsberg 45/1 · 89081 Ulm · Germany
Phone +49 (0)731 500-63101 · Fax +49 (0)731 500-63082 · www.euro-hd.net

EUROPEAN HUNTINGTON'S DISEASE NETWORK

Lisbon-Central (Hospital dos Capuchos, Centro Hositalar Lisboa Central): Manuel Almeida, Ana Calado, Margarida Dias, Joana Morgado, Cristina Semedo

Lisbon-HSM (Hospital de Santa Maria, Clinical Pharmacology Unit, Instituto de Medicina Molecular): Leonor Correia Guedes, Miguel Coelho, Joaquim J Ferreira, Andreia Magalhães, Tiago Mestre, Tiago Mendes, Dulce Neutel, Filipe Rodrigues, Anabela Valadas

Lisbon-HFF (Hospital Fernando da Fonseca): Cristina Costa, Helena Cardoso, Tiago Mendes, Mariana Santos

Porto-HGSA (Hospital Santo António- Centro Hospitalar do Porto): Gonçalo Cação, Sara Cavaco, Joana Damásio, Joana Fernandes, Alexandra Gonçalves, Rui Loureiro, Inês Moreira, Marina Magalhães, Paula Salgado

Porto- HSJ (Hospital de São João): Carlos Andrade, Andreia Costa, Carolina Garrett, Miguel Gago, Joana Guimarães, João Massano, Joana Meireles, Ana Monteiro

RUSSIAN FEDERATION

Kazan: Diana Khasanova, Zuleykha Zalyalova

Moscow – (Research Center of Neurology): Sergey Illarioshkin, Sergey Klyushnikov, Olga Sidorova, Oleg Smirnov, Elizaveta Yudina, Yury Seliverstov

Nizhny Novgorod – (Nizhny Novgorod Medical Academy, Neurology Department): Victoria Antonova, Svetlana Kopishinskaya, Maria Korotysh

Ufa – (Bashkir State Medical University, Department of Neurology, Neurosurgery, and Medical Genetics): Rim Magzhanov, Elena Saifullina

Voronezh: Sergey Kurbatov

SPAIN

Alicante-Alcoy (Hospital Virgen de los Lirios): Pilar Solis

Badajoz (Hospital Infanta Cristina): Carmen Durán Herrera, Patrocinio Garcia Moreno

Barcelona-Bellvitge (Hospital Universitari de Bellvitge): Jordi Bas, Núria Busquets, Matilde Calopa, Serge Jaumà Classen, Nadia Rodríguez Dedichá

Barcelona- Clínic i Provincial (Hospital Clínic i Provincial): María Teresa Buongiorno, Andrés de la Cerda Santa María, Esteban Muñoz, Pilar Santacruz

Barcelona-Hospital Mútua de Terrassa: Miquel Aguilar Barbera, Ana Rojo Sebastián, Sonia Arribas Pardo, Dolors Badenes Guia, Noemi Calzado, Laura Casas Hernanz, Juan Pablo Tartari Díaz-Zorita, Judit López Catena, Pilar Quiléz Ferrer, Gemma Tome Carruesco

Barcelona-Merced (Hospital Mare de Deu de La Merced): Misericordia Floriach Robert, Cèlia Mareca Viladrich, Elvira Roca, Jesús Miguel Ruiz Idiago, Antonio Villa Riballo

Barcelona-Santa Cruz y San Pablo (Hospital de la Santa Creu i Sant Pau): Antonia Campolongo, Ramon Fernandez de Bobadilla, Andrea Horta, Jaime Kulisevsky Bojarsky, Saul Martinez-Horta, Javier Pagonabarraga, Jesus Perez Perez, Roser Ribosa, Carolina Villa

Bilbao (Hospital de Cruces): Maria Angeles Acera Gil, Koldo Berganzo Corrales, Juan Carlos Gomez Esteban, Amaia González, Beatriz Tijero Merino

Burgos (Servicio de Neurología Hospital General Yagüe): Esther Cubo, Cecilia Gil Polo, Natividad Mariscal, Jesús Sánchez

Oberer Eselsberg 45/1 · 89081 Ulm · Germany
Phone +49 (0)731 500-63101 · Fax +49 (0)731 500-63082 · www.euro-hd.net

Canarias (Hospital Insular de Gran Canaria): Sandra Gutierrez Romero, José Matías Arbelo, Rocío Malo de Molina, Idaira Martín, Juan Manuel Periañez, Beatriz Udaeta

Fuenlabrada (Hospital Universitario): Fernando Alonso-Frech, María del Valle Loarte

Granada (Hospital Universitario San Cecilio, Neurología): Francisco Barrero, Blas Morales

Madrid-BTCIEN (Fundación CIEN): Belén Frades, Marina Ávila Villanueva, Maria Ascension Zea Sevilla

Madrid-Clínico (Hospital Clínico Universitario San Carlos): María del Mar Fenollar, Rocío García-Ramos García, Clara Villanueva

Madrid RYC (Hospital Ramón y Cajal, Neurología): Mónica Bascuñana, Marta Fatás Ventura, Juan García Caldentey, Guillermo García Ribas, Justo García de Yébenes, José Luis López–Sendón Moreno, Verónica Mañanes Barral, Patricia Trigo Cubillo

Madrid FJD (Madrid-Fundación Jiménez Díaz): Cici Feliz, Pedro José García Ruíz, Ana García, Juan García Caldentey, Rosa Guerrero López, Antonio Herranz Bárcenas, Asunción Martínez-Descals, Angel Martínez Pueyo, Veronica Puertas Martin, Noelia Rodríguez Martínez, Teresa Montojo, María José Sainz Artiga, Vicenta Sánchez

Murcia (Hospital Universitario Virgen de la Arrixaca): María Dolores Alarcón, Carmen Antúnez Almagro, Esther Diéguez, Lorenza Fortuna, Agustina Legaz, Salvadora Manzanares, Juan Marín Muñoz, María Martirio Antequera Torres, Fuensanta Noguera Perea, Laura Vivancos

Oviedo (Hospital Central de Asturias): Sonia González, Luis Menéndez Guisasola, Marta Para Prieto, René Ribacoba, Carlos Salvador, Pablo Sánchez Lozano

Palma de Mallorca (Hospital Universitario Son Espases): Juan García Caldentey, Inés Legarda Ramirez, Dolors Moragues Benito, Penelope Navas Arques, Monica Rodriguez Lopera, Barbara Vives Pastor

Pamplona (Complejo Hospitalario de Navarra): Itziar Gaston, Fermin Garcia-Amigot, Maria Dolores Martinez-Jaurrieta, Maria Antonia Ramos-Arroyo

Sevilla (Hospital Universitario Virgen del Rocío): Astrid Adarmes, Maravilla Bernal-Escudero, Fátima Carrillo, Silvia Jesús, Pablo Mir, Laura Vargas-González

Sevilla (Hospital Virgen Macarena): Fátima Damas Hermoso, José Manuel García Moreno, Javier Abril Jaramillo, Carolina Mendez Lucena, Eva María Pacheco Cortegana, José Chacón Peña, Luis Redondo, Violeta Sánchez Sánchez

Sevilla (Residencia Santa Ana): Cristina Melgar Fernandez, María Dolores Romero Lemos, Maite Paredes Mata, Rocío Villagrán Casado

Valencia (Hospital la Fe): Maria Bosca, Juan Andres Burguera, Francisco Castera Brugada, Jose Maria Millán Salvador, Carmen Peiró Vilaplana, Pilar Solís, Begoña Jeweinat Figuerola, Paloma Millan Palanca

Zaragoza (Hospital Clínico): Elena Bellosta Diago, Javier López del Val, Laura Martinez Martinez, Elena López

SWEDEN

Göteborg (Sahlgrenska University Hospital): Jan Wahlström+, Ulrika Høsterey-Ugander, Gunnel Fredlund, Radu Constantinescu, Kajsa Lewin, Liselotte Neleborn-Lingefjärd, Maria Berglund, Peter Berglund, Petra Linnsand

Oberer Eselsberg 45/1 · 89081 Ulm · Germany
Phone +49 (0)731 500-63101 · Fax +49 (0)731 500-63082 · www.euro-hd.net

Lund (Dept Neurology, Skånes Universityhospital): Åsa Petersén, Jan Reimer, Håkan Widner

Stockholm-Ersta: Mouna Esmaeilzadeh, Joakim Tedroff, Elisabeth Winnberg

Stockholm Karolinska University Hospital: Stanislav Benaminov, Elisabeth Björnsson, Daniel Merrick, Martin Paucar, Sven Pålhagen, Per Svenningsson, Tina Wallden

Umeå (Umeå University Hospital): Måns Berglund, Ghada Loutfi, Carina Olofsson, Eva-Lena Stattin, Laila Westman, Birgitta Wikström

Uppsala University Hospital: Camilla Ekwall, Marie-Lousie Göller, Anders Johansson, Valter Niemelä, Dag Nyholm, Jimmy Sundblom, Leif Wiklund

SWITZERLAND

Bern: Jean-Marc Burgunder, Jessica Koehli, Yanik Stebler (Swiss HD Zentrum), Alain Kaelin, Irene Romero, Michael Schüpbach, Sabine Weber Zaugg (Zentrum für Bewegungsstörungen, Neurologische Klinik und Poliklinik, Universität Bern)

Lausanne: Federica Esposito, Jean-Marc Good, Karin Paus, Francois Vingerhoets, Christian Wider+

Zürich (University Hospital and University of Zurich): Hans H. Jung, Jens A. Petersen, Maria Ligon-Auer, Violeta Mihaylova

U.K.

Aberdeen (NHS Grampian Clinical Genetics Centre & University of Aberdeen): Lorna Downie, Roisin Jack, Kirsty Matheson, Zosia Miedzybrodzka, Daniela Rae, Sheila A Simpson, Fiona Summers, Alexandra Ure, Vivien Vaughan

Barnstaple: Timothy Harrower, Nathan Vernon

Birmingham (The Barberry Centre, Dept of Psychiatry): Shahbana Akhtar, Jenny Crooks, Adrienne Curtis, Jenny de Souza (Keylock), John Piedad, Hugh Rickards, Jan Wright

Blanford Forum: Diane Haig-Brown, Janet Craven, Andrew Pallett, Steve Simpson, Rebecca Weekes

Bristol (North Bristol NHs Trust, Southmead hospital): Elizabeth Coulthard, Louise Gethin, Beverley Hayward, Kasia Sieradzan, Abigail Wright

Cambridge (Cambridge Centre for Brain Repair, Forvie Site): Roger A. Barker, Deidre O'Keefe, Anna Gerrtiz (nee Di Pietro), Kate Fisher, Anna Goodman, Susan Hill, Sarah Mason, Rachel Swain, Natalie Valle Guzman

Cardiff (Schools of Medicine and Biosciences, Cardiff University): Jonathan Bisson, Monica Busse, Cynthia Butcher, Rebecca Cousins, Jenny Callaghan, Stephen Dunnett, Catherine Clenaghan, Ruth Fullam, Sarah Hunt, Lesley Jones, Una Jones, Hanan Khalil, Sara Minster, Michael Owen, Kathleen Price, Jenny Townhill, Anne Rosser

Dundee (Scottish Huntington's Association, Ninewells Hospital): David Goudie, Lindsay Buchanan, Paula McFadyen, Alison Tonner, Anne-Marie Taylor

Edinburgh (SE Scotland Genetic Service, Western General Hospital): Maureen Edwards, Carrie Ho (Scottish Huntington´s Association), Marie McGill, Mary Porteous, Pauline Pearson

Oberer Eselsberg 45/1 · 89081 Ulm · Germany
Phone +49 (0)731 500-63101 · Fax +49 (0)731 500-63082 · www.euro-hd.net

Exeter (Department of Neurology Royal Devon and Exeter Foundation Trust Hospital): Timothy Harrower, Sarah Irvine

Fife (Scottish Huntington's Association Whyteman's Brae Hospital): Peter Brockie, Jillian Foster, Nicola Johns, Sue McKenzie, Jean Rothery, Gareth Thomas, Shona Yates

Forth_Valley (Neurology Department, Forth Valley Royal Hospital): Christian Neumann, Kirsten Patterson, David Thomson

Glasgow (Glasgow HD Management Clinic, Southern General Hospital): Catherine Deith, Jane Ireland, Stuart Ritchie

Gloucester (Department of Neurology Gloucestershire Royal Hospital): Pauline Brown, Liz Burrows, Amy Fletcher, Alison Harding, Kaye Harrison, Fiona Laver, Mark Silva, Aileen Thomson

Hull (Castle Hill Hospital): Carol Chu, Carole Evans, Deena Gallentree, Stephanie Hamer, Alison Kraus, Ivana Markova, Ashok Raman, Liz Rowett

Launceston (Millaton Court): Alyson Andrew, Julie Frost, Rupert Noad

Leeds (Chapel Allerton Hospital, Department of Clinical Genetics): Jeremy Cosgrove, Deena Gallantree, Stephanie Hamer, Emma Hobson, Stuart Jamieson , Alison Kraus, Mandy Longthorpe, Ivana Markova, Hannah Musgrave, Caroline Peacy, Ashok Raman, Liz Rowett, Jean Toscano, Sue Wild, Pam Yardumian

Leicester (Leicestershire Partnership Trust, Mill Lodge): Carole Clayton, Heather Dipple, Dawn Freire-Patino, Caroline Hallam, Julia Middleton

Liverpool (Walton Centre for Neurology and Neurosurgery): Sundus Alusi, Rhys Davies, Kevin Foy, Emily Gerrans, Louise Pate

London (St. Georges-Hospital): Uruj Anjum, Jan Coebergh, Charlotte Eddy, Nayana Lahiri, Meriel McEntagart, Michael Patton, Maria Peterson, Sarah Rose

London (Guy's Hospital): Thomasin Andrews, Andrew Dougherty, Charlotte Golding, Fred Kavalier, Hana Laing, Alison Lashwood, Dene Robertson, Deborah Ruddy, Alastair Santhouse, Anna Whaite

London (The National Hospital for Neurology and Neurosurgery): Thomasin Andrews, Stefanie Gosling (nee Brown), Stefania Bruno, Elvina Chu, Karen Doherty, Charlotte Golding, Salman Haider, Davina Hensman, Nayana Lahiri, Monica Lewis, Marianne Novak, Aakta Patel, Nicola Robertson, Elisabeth Rosser, Sarah Tabrizi, Rachel Taylor, Thomas Warner, Edward Wild

London (Royal Hospital for Neuro-disability): Oda Ackermann, Sophie Duport, Adrienne Scott, Nicholas Stoy, Jenny Vaughn

Manchester (Genetic Medicine, University of Manchester, Manchester Academic Health Sciences Centre and Central Manchester University Hospitals NHS Foundation Trust): Natalie Arran, Judith Bek, Jenny Callaghan, David Craufurd, Ruth Fullam, Marianne Hare, Liz Howard, Susan Huson, Liz Johnson, Mary Jones, Ashok Krishnamoorthy, Helen Murphy, Emma Oughton, Lucy Partington-Jones, Dawn Rogers, Andrea Sollom, Julie Snowden, Cheryl Stopford, Jennifer Thompson, Iris Trender-Gerhard, Nichola Verstraelen (formerly Ritchie), Leann Westmoreland

Newcastle-upon-Tyne (Centre for Life, Institute of Medical Genetics): Ginette Cass, Lynn Davidson, Jill Davison, Neil Fullerton, Katrina Holmes, Suresh Komati, Sharon McDonnell, Zeid Mohammed, Karen Morgan, Lois Savage, Baldev Singh, Josh Wood

Northampton (St Andrew’s Healthcare): Elvina Chu, Caroline Knight, Mari O’Neill, Debasish Das Purkayastha

Oberer Eselsberg 45/1 · 89081 Ulm · Germany
Phone +49 (0)731 500-63101 · Fax +49 (0)731 500-63082 · www.euro-hd.net

Oxford (Oxford University Hospitals NHS Trust, Dept. of Neurosciences, University of Oxford): Andrea H Nemeth, Gill Siuda, Ruth Valentine, Kathryn Dixon, Richard Armstrong

Plymouth (Plymouth Huntington Disease Service, Mount Gould Hospital): David Harrison, Max Hughes, Sandra Large, John O Donovan, Amy Palmer, Andrew Parkinson, Beverley Soltysiak, Leanne Timings, Josh Williams

Poole (Brain Injury Service, Poole Hospital): John Burn, Rebecca Weekes, Janet Craven, Wendy Bailey, Caroline Coleman, Diane Haig-Brown, Steve Simpson

Preston (Neurology Department, Preston Royal Hospital): Marianne Hare, Tahir Majeed, Nicola Verstraelen (Ritchie)

Reading (Royal Berkshire Hospital): Richard Armstrong, Kathryn Dixon, Wendy Barrett, Aileen Ho

Sheffield (The Royal Hallamshire Hospital– Sheffield Children's Hospital): Oliver Bandmann, Alyson Bradbury, Helen Fairtlough, Kay Fillingham, Isabella Foustanos, Paul Gill, Mbombe Kazoka, Kirsty O'Donovan, Louise Nevitt, Nadia Peppa, Oliver Quarrell, Cat Taylor, Katherine Tidswell

Southampton (Southampton General Hospital): Christopher Kipps, Lesley MacKinnon, Veena Agarwal, Elaine Hayward, Kerry Gunner, Kayla Harris, Mary Anderson, Melanie Heywood, Liane Keys, Sarah Smalley

Stoke on Trent (Bucknall Hospital): George El-Nimr, Allison Duffell, Sue Wood, Karen Kennedy (nee Smith)

Swindon (Victoria Centre, Great Western Hospital): Lesley Gowers, Kingsley Powell, Pamela Bethwaite, Rachel Edwards, Kathleen Fuller, Michelle Phillips

EHDN's associate site in Singapore:

National Neuroscience Institute Singapore: Louis Tan, Jean-Marc Burgunder, Puay Ngoh Lau, Emmanuel Pica

Oberer Eselsberg 45/1 · 89081 Ulm · Germany
Phone +49 (0)731 500-63101 · Fax +49 (0)731 500-63082 · www.euro-hd.net
